# Supplementary material for: Evidence for an adverse impact of remote readouts on radiology resident productivity: Implications for training and clinical practice
Source: PLOS Digit Health. 2023 Sep 22;2(9):e0000332. doi: 10.1371/journal.pdig.0000332 (PMC10516412; doi:10.1371/journal.pdig.0000332)
Supplement: S1 Table — Pre-pandemic encompasses July 1st through December 31st 2018 and 2019 and is compared to the pandemic period during July 1st through December 31st of 2020. Two-sided t-test, * denotes statistically significant p values<0.05. (DOCX) [file pdig.0000332.s001.docx]

| **S1 Table—**Mean number of resident co-authored studies per rotation, by readout model and resident year. | | | | | | | | | | | | | | | | | | | | | | | | |  |
| --- | --- | --- | --- | --- | --- | --- | --- | --- | --- | --- | --- | --- | --- | --- | --- | --- | --- | --- | --- | --- | --- | --- | --- | --- | --- |
|  | **Division readout model** | | | | | | | | | | | | | | | |  | | |  |  | |  |  |  |
|  | **Hybrid** | | |  |  | | | **Remote** | |  |  | | | **All divisions** | | | |  | | |  |  |  |  |  |
|  | pre-pandemic  (N) [CI] | pandemic  (N) [CI] | *p* | | |  | pre-pandemic  (N) [CI] | | pandemic  (N) [CI] | | | *p* |  | | pre-pandemic  (N) [CI] | pandemic  (N) [CI] | | | *p* | |  |  |  |  |  |
| All | 212.4 (255)  [195.2-233.8] | 209.6 (130)  [180.3-238.9] | .81 | | |  | 300.5 (114)  [258.8-342.2] | | 229.4 (52)  [183.3-275.5] | | | .043* |  | | 239.6 (369)  [222.2-260.0] | 215.3 (182)  [190.8-239.8] | | | .13 | |  |  |  |  |  |
| R1 | 219.7 (85)  [180.5-258.9] | 203.8 (46)  [161.1-246.5] | .61 | | |  | 268.9 (38)  [232.6-305.2] | | 203.5 (21)  [160.8-246.2] | | | .026* |  | | 234.9 (123)  [205.6-264.2] | 203.7 (67)  [172.1-235.3] | | | .18 | |  |  |  |  |  |
| R2 | 213.0 (80)  [184.6-241.3] | 180.7 (35)  [146.4-215.1] | .18 | | |  | 357.8 (41)   [278.9-436.7] | | 251 (16)  [167.3-334.7] | | | .12 |  | | 262.0 (121)  [227.8-296.3] | 202.8 (51)  [168.0-237.6] | | | .042* | |  |  |  |  |  |
| R3 | 210.9 (90)  [167.4-295.8] | 235.7 (49)  [167.4-295.8] | .44 | | |  | 267.9 (35)  [173.8-361.9] | | 242.6 (15)  [107.9-377.3] | | | .76 |  | | 226.9 (125)  [192.1-261.6] | 237.3 (64)  [181.3-293.3] | | | .74 | |  |  |  |  |  |
| Pre-pandemic encompasses July 1^st^ through December 31^st^ 2018 and 2019 and is compared to the pandemic period during July 1^st^ through December 31^st^ of 2020. Two-sided *t*-test, * denotes statistically significant p values<0.05. | | | | | | | | | | | | | | | | | | | | | | | | |  |
|  | |  |  |  |  |  |  |  |  |  |  |  |  |  |  |  |  |  |  |  |  |  |  |  |  |
