# Supplementary material for: Evidence for an adverse impact of remote readouts on radiology resident productivity: Implications for training and clinical practice
Source: PLOS Digit Health. 2023 Sep 22;2(9):e0000332. doi: 10.1371/journal.pdig.0000332 (PMC10516412; doi:10.1371/journal.pdig.0000332)
Supplement: S2 Table — †pre: the period before the pandemic encompasses July 1st through December 31st 2018 and 2019 and is compared to the pandemic period July 1st through December 31st 2020. Two-sided t-test, * denotes statistically significant p values<0.05. FL: Fluoro, MG: Mammography, XR: Radiographs, US: Ultrasound, NM: Nuclear Medicine, CT/MR: Cross-sectional studies. (DOCX) [file pdig.0000332.s002.docx]

| **S2 Table.** Percent composition of imaging modalities for all rotations and the remote divisions. | | | | | | | | | | | | | | | | | | | |  |  |  |
| --- | --- | --- | --- | --- | --- | --- | --- | --- | --- | --- | --- | --- | --- | --- | --- | --- | --- | --- | --- | --- | --- | --- |
|  | **All rotations** | | |  | | |  | **Thoracic radiology** | | | |  | | **Pediatric radiology** | | | | |  | | | |
|  | †pre- | pandemic | *p* | |  | pre- | | | pandemic | *p* |  | | pre- | | pandemic | *p* |  | | | |  |  |
| CT/MR | 34.8% | 37.3% | 0.45 | |  | 39.3% | | | 36.2% | .74 |  | | 9.1% | | 7.4% | .50 |  | | | |  |  |
| XR | 35.3% | 33.1% | 0.53 | |  | 60.7% | | | 63.8% | .74 |  | | 77.9% | | 70.4% | .04* |  | | | |  |  |
| US | 12.4% | 11.4% | 0.58 | |  | 0 | | | 0 |  |  | | 9.9% | | 18.4% | <.001* |  | | | |  |  |
| FL | 2.8% | 3.2% | 0.53 | |  | 0 | | | 0 |  |  | | 3.0% | | 3.7% | .62 |  | | | |  |  |
| MG | 6.1% | 6.8% | 0.69 | |  | 0 | | | 0 |  |  | | 0 | | 0 |  |  | | | |  |  |
| NM | 8.6% | 8.2% | 0.85 | |  | 0 | | | 0 |  |  | | <0.1% | | 0 | .32 |  | | | |  |  |
| †pre: the period before the pandemic encompasses July 1^st^ through December 31^st^ 2018 and 2019 and is compared to the pandemic period July 1^st^ through December 31^st^ 2020. Two-sided *t*-test, * denotes statistically significant p values<0.05. FL: Fluoro, MG: Mammography, XR: Radiographs, US: Ultrasound, NM: Nuclear Medicine, CT/MR: Cross-sectional studies | | | | | | | | | | | | | | | | | |  | | | |  |
