# Supplementary material for: Evidence for an adverse impact of remote readouts on radiology resident productivity: Implications for training and clinical practice
Source: PLOS Digit Health. 2023 Sep 22;2(9):e0000332. doi: 10.1371/journal.pdig.0000332 (PMC10516412; doi:10.1371/journal.pdig.0000332)
Supplement: S3 Table — † Cross terms are denoted with • between interacting variables. p values of < .05, < .01, and < .001 are denoted with *,**,***, respectively. Pre: pre-pandemic period; Pand: pandemic period between July 1st and December 31st, 2020. FL: Fluoro; MG: Mammography; XR: Radiographs; US: Ultrasound, NM: Nuclear Medicine, CT/MR: Cross-sectional studies. (DOCX) [file pdig.0000332.s003.docx]

|  | |  |  |
| --- | --- | --- | --- |
| **S3 Table.** Multivariate regression by resident level | | | |
| Variable † | R1 | R2 | R3 |
| Pre•Remote | reference | reference | reference |
| Pandemic•Remote | -72.3** | -101.8 | -65.8 |
| Pre•Hybrid | 17.2 | -99.23* | 30.8 |
| Pandemic•Hybrid | 1.1 | -122.5* | 43.8 |
|  |  |  |  |
| *Modality* |  |  |  |
| Radiography | reference | reference | reference |
| CT/MR | -91.2*** | -56.9 | -223.6*** |
| Fluoro | 723.4*** | 163.5 | -1558.5*** |
| Mammo | -300.7*** | -197.8*** | -355.0*** |
| NM | -69.2** | -158.5*** | -340.5*** |
| US | 265.9*** | -70.3 | 167.2* |
| Constant | 225.8 | 369.9 | 361.4 |
| *R*^2^ | 0.483 | 0.218 | 0.343 |
| *F* stat | <0.0001 | <0.0001 | <0.0001 |
| # of Obs | 190 | 172 | 189 |
| † Cross terms are denoted with • between interacting variables. *p* values of <.05, <.01, and <.001 are denoted with *,**,***, respectively. Pre: pre-pandemic period; Pand: pandemic period between July 1^st^ and December 31^st^, 2020. FL: Fluoro; MG: Mammography; XR: Radiographs; US: Ultrasound, NM: Nuclear Medicine, CT/MR: Cross-sectional studies | | | |
