# Supplementary material for: Evidence for an adverse impact of remote readouts on radiology resident productivity: Implications for training and clinical practice
Source: PLOS Digit Health. 2023 Sep 22;2(9):e0000332. doi: 10.1371/journal.pdig.0000332 (PMC10516412; doi:10.1371/journal.pdig.0000332)
Supplement: Attachment — Submitted filename: Response to Reviewers.docx [file pdig.0000332.s004.docx]

# Editor comments:

1. I assume the abstract should be unstructured and shorter based on the journal instructions. We have shortened the abstract, and it is now unstructured.

2. In the abstract, please revise the aim of the study to make it clearer. It should be consistent with the aim written in the introduction section.

Thank you for pointing this out. We fixed this so the aim of the study is clearer and consistent between the abstract and introducation.

3. In the methods section, please use appropriate subheadings to explain research setting, participants, sampling, research instrument, data collection and analysis, ethics approval, etc. Done.
4. Please remove the footers which show different sections of the manuscript.

Done.

5. Please improve the readability of the manuscript to avoid any possible typos, unclear/ complex sentences, etc.

We have reworded confusing portions of the text.

6. In the discussion section, please provide possible reasons for interpreting fewer studies by radiology residents during remote rotations. We have expanded upon these reasons in a new paragraph within the discussion section. Please see paragraphs 5 and 6 of the discussion section.

# Reviewer 1:

## Major

**1a. Reviewer.** The authors have categorised the sub-specialty departments based on their remote vs hybrid practice during the pandemic, however it’s unclear what the practice was in these departments pre-pandemic. Was there a change in the extent of remote reporting in 2020? This is of particular relevance for the thoracic and paediatric departments – if the authors can demonstrate that there was a change in practice pre-pandemic vs pandemic, this would strengthen the link between the two and limit the possibility of other confounders accounting for the results. Also related to this, how was it determined where senior faculty were reporting from for each six month block?

**1b. Author response:**

We have reworded portions of the text for further clarification. Prior to the COVID-19 pandemic, the standard practice involved on-site residents and attendings and shoulder-to-shoulder readouts; remote readouts were essentially non-existent at our institution. With the onset of the pandemic in 2020, readouts were variably introduced to all divisions. Therefore, 'remote' divisions had a complete change in their readout practice, whereas 'hybrid' divisions had a mix of on-site and remote faculty.

We selected the six-month period for two reasons: 1) Our institution's volumes returned to normal after the steep initial decline, and 2) We could not compare remote versus hybrid divisions after this period because the remote divisions began bringing faculty back on-site. It's worth noting that all residents worked on-site during this period.

No data was collected during the pandemic for specifics on the proportion of attendings and readouts that were on-site versus remote in the hybrid divisions. However, we recommend in our paper that residencies should closely monitor the location and timing of remote work in the future to more specifically understand the effect on resident education and case volumes.

**2a. Reviewer**.

The results are interesting, however the discussion lacks explanation as to why this drop in resident reporting might be. In practice, do residents normally report a study, then save the report/release a preliminary report to the clinicians until the attending reviews the report and finalises it with any necessary changes? Do they have to speak to the attending for each report? It would be useful to clarify how this works logistically in the authors’ department in the manuscript. If resident’s simply save/release preliminary reports and wait for them to be reviewed, why do the authors think the residents are reporting less in 2020 on remote rotations?

**2b. Author response.**

Thank you. We have added a new paragraph in the discussion section that clarifies the text and discusses these points.

## Minor

**1a. Reviewer.** Consider adding radiology as a keyword.

**1b. Author response**. Added.

**2a. Reviewer.** Although this is clarified later on in the manuscript, it is unclear in the abstract which group is working remotely. As it stands, it could be interpreted as residents working remotely instead of the attendings.

**2b. Author response.** We rephrased the abstract to clarify this point.

**3a. Reviewer.** I have been asked to comment on the availability of the raw data, but I could not find/did not have access to this during my review.

**3b**. **Author response.** The data can be accessed for replication in the Harvard Dataverse at the following link: https://dataverse.harvard.edu/privateurl.xhtml?token=148db9b2-e55f-4fe6-b26d-19b645783217

**4a. Reviewer.** Can the authors briefly explain what wRVUs are in the manuscript. Most non-US radiologists will not be familiar with this term.

**4b. Author response.** We have clarified the meaning and general purpose of wRVUs in the methods section. It is a national standardized parameter used to estimate physician productivity for billing purposes.

**5a. Reviewer.**

Why did the authors group CT and MR into one group instead of two? Also, I don’t think either of these terms were spelled out before acronyms were introduced

**5b. Author response.** CT and MRI were grouped because these are cross-sectional modalities, unlike the other studies evaluated, and it is not uncommon to do so within the radiology literature. Both modalities are similar in that, compared to the other modalities, they are much fewer in number, more complex, and take much longer to interpret. We have clarified the abbreviations for computed tomography and magnetic resonance, and several other modalities in the methods section.

**6a. Reviewer.** Results line 144: It’s confusing that the authors are investigating 2018-2020 but it mentions here that data was investigated over 18 months?

**6b. Author response.** Three six-month periods for each of the three years were examined (a total of 18 months). We agree that the text was unnecessary and confusingly worded. We simplified it to "all the months studied."

**7a. Reviewer.** Results line 152: “Departmental wRVUs in 2020 (463,781) were 92% of the 2018 and 2019 average (477,179)”. 463781/477179 = 97% not 92%.

**7b. Author response.** We corrected this in the text. Additionally, three minor typographical/keyboarding errors were corrected in Tables 1 and 2. For example, the number of studies was listed as 128,817, which was corrected to 128,187.

**8a. Reviewer.** Results line 159: Which half years of 2018 and 2019 are these figures referring to? In the following sentences it’s also unclear which halves of 2018, 2019 and 2020 are being compared.

**8b. Author response.** We have modified parts of the text and figure legends to clarify that all periods evaluated were between July 1 to December 31st for all years. The period evaluated for all years is also described at the beginning of the methods and results section.

**9a. Reviewer.** Can the authors confirm that there was no significant change in the attending staff of the departments which could serve as a confounder?

**9b. Author response.** At our institution, fluctuations in faculty numbers are unlikely to impact resident productivity significantly. This is supported by the fact that at our institution, attending radiologists interpret most imaging studies (>50%) independently of residents.

We did observe minor variations in faculty numbers across divisions, but they didn't follow a consistent pattern and occurred at inconsistent intervals, making it too complex to include in the regression model. Nevertheless, it's worth noting that even with an increase of two faculty members in the remote pediatric division and a decrease of one in the thoracic division, both saw a decline in resident productivity. Meanwhile, some hybrid divisions like MSK and AI, despite losing more faculty (1 and 2, respectively), maintained their productivity levels.

These observations, particularly during the transition to remote work, strongly suggest that faculty numbers did not significantly confound our results, thus affirming our initial regression model.

# Reviewer 2:

**1a. Reviewer.** An overview of the effect of telemedicine in general and remote health in the covid era is necessary in the introduction before introduction residence remote interpretation for a better flow of the manuscript. a good paper to select info from: Telemedicine in the COVID-19 Era: A Narrative Review Based on Current Evidence. Int J Environ Res Public Health. 2022 Apr 22;19(9):5101. doi: 10.3390/ijerph19095101;

**1b. Author response.**	We appreciate the suggestion and have integrated the reference provided. This enhances our manuscript's scope by underscoring the far-reaching impacts of the telemedicine shift across various medical disciplines. This will also ensure that the reader recognizes the broader context and understands how the continued transition to digital health underpins our discussion on residential remote interpretation.

**2a. Reviewer.** an explanation why the radiology residences and not other specialisation was selected for this study

**2b. Author response.** The shift to remote operations instigated substantial and immediate changes to the workflow of radiology departments, in particular, it disrupted the traditional in-person readouts. Radiology residencies, where case volumes are integral to education, are uniquely susceptible to these alterations. Furthermore, as all authors involved in this study are practicing radiologists, our firsthand experiences lent us deeper insights into these specific changes. We have clarified portions of the text to underscore this rationale.

**3a. Reviewer.** and/or if a correlation with other specialisations in regards to tele-learning is of valid proposal for future studies.

**3b. Author response.** We have incorporated the review article mentioned in 1a into our introduction, which clearly demonstrates the broad-ranging impact of remote work across various specialties. Based on our findings, we fully concur that studying the impact of telelearning on trainees in other specialties would be of great importance for their respective fields

**4a. Reviewer.** if possible, a correlation of board pass rate and residence case volumes for this period should be introduced in the manuscript if available.

**4b. Author response.** This is a fantastic idea and a promising subject for future exploration. We have mentioned the importance of this in the text previously: "It is important to continue tracking residents affected by the pandemic to better understand if there are differences in board exam preparedness or clinical acumen"
